# Fragility Femoral and Hip Fractures in Saudi Arabia: A Systematic Review of Epidemiology, Risk Factors, Clinical Outcomes, and Health-System Gaps

**DOI:** 10.7759/cureus.100266

**Published:** 2025-12-28

**Authors:** Mohaned S Argan, Hatem Soliman Ali Alwalidi, Lamar Tariq Alassiry, Maha Ali Alturki, Lamya Ghanim Aldaraani, Adel Ali Asiri, Mohammed Y Asiri, Arwa Mudawi Asiri, Wafaa Sulaiman Alhifzi, Refal F Faya, Mohammad Hassan I Ahmasani, Abdulrhman Saeid Alshahrani, Lama Nasser Alqahtani, Saad Dhafer Alshahrani

**Affiliations:** 1 Orthopedics, Armed Forced Hospitals Southern Region, Khamis Mushait, SAU; 2 General Surgery, Aseer Central Hospital, Abha, SAU; 3 General Surgery, King Khalid University, Abha, SAU; 4 Orthopedic Surgery, Aseer Central Hospital, Abha, SAU; 5 Orthopedic Surgery, Saudi German Hospital, Jeddah, SAU; 6 Medicine, King Khalid University, Abha, SAU; 7 General Practice, Aseer Central Hospital, Abha, SAU; 8 General Practice, College of Medicine, King Khalid University, Abha, SAU; 9 General Surgery, University of Bisha, Bisha, SAU

**Keywords:** epidemiology, femoral fracture, hip fracture, risk factors, saudi arabia

## Abstract

Fragility hip and femoral fractures are a major cause of morbidity, mortality, and healthcare utilization, yet evidence from Saudi Arabia remains fragmented. This systematic review with narrative synthesis evaluated studies conducted in Saudi Arabia that examined the incidence, risk factors, clinical outcomes, and health-system gaps associated with fragility hip and femoral fractures among adults aged 50 years and older. Searches of PubMed, Scopus, Web of Science, and Google Scholar identified 13 eligible studies published between 2015 and 2025. Across predominantly hospital-based cohorts, hip and femoral fractures accounted for a substantial proportion of orthopedic admissions. Estimates of national fracture burden were derived from individual modeling studies and suggest more than 7,000 femoral fractures annually. Reported one-year mortality varied widely across studies, ranging from approximately 11% to nearly 48%, with higher mortality consistently observed among patients with delayed surgery or higher ASA classification. Functional recovery was frequently limited, and postoperative complications were common. Despite a high prevalence of osteoporosis or osteopenia among fracture patients, osteoporosis diagnosis and secondary prevention were consistently underutilized. Dual-energy X-ray absorptiometry (DEXA) scanning was reported in fewer than 20% of patients, and most individuals received no post-fracture osteoporosis therapy. Studies also highlighted variability in clinical practice, low public awareness, and gaps in postoperative mobilization and follow-up care. Although the evidence base is limited and heterogeneous, the included studies consistently identify delayed surgical care, underdiagnosis and undertreatment of osteoporosis, and system-level practice variability as key challenges in the Saudi context. These findings support the need for locally adapted strategies that prioritize timely surgery, standardized care pathways, and improved secondary fracture prevention.

## Introduction and background

Osteoporosis and its resulting fragility fractures represent a major global health concern, particularly among aging populations [1]. As bone mass declines with age and neuromuscular function weakens, the risk of fractures rises sharply, especially in postmenopausal women [1]. Hip fractures are the most severe outcome of osteoporosis because they are strongly linked to low bone mineral density, almost always require hospitalization, and lead to substantial disability and mortality [2]. Worldwide, an estimated nine million osteoporotic fractures occurred in the year 2000, including 1.6 million hip fractures, reflecting a considerable clinical and economic burden [2]. In Europe alone, fragility fractures accounted for more than 37 billion euros in direct medical costs in 2010, a figure projected to increase by 25 percent by 2025 [3]. Similarly, in the United States, over two million osteoporosis-related fractures were recorded in 2005 with associated costs exceeding 17 billion dollars, and projections indicate a major rise in fracture incidence as populations age [4].

Hip fracture incidence increases exponentially with age in both sexes. Women experience nearly twice the rate of hip fractures as men due to greater age-related bone loss and a higher prevalence of falls [5]. Geographic variability is also well documented, with some regions reporting incidence rates several times higher than others [6]. Such differences reflect variations in bone health, physical activity, environmental conditions, and population structure.

In Saudi Arabia, osteoporosis and fragility fractures constitute a rising public health issue. Earlier epidemiological work reported osteoporosis prevalence ranging between 30 and 48 percent, which appears comparable to or higher than many Western populations [7]. A 2012 systematic review estimated that more than one-third of healthy Saudi women aged 50 to 79 years were osteopenic and another one-third were osteoporotic [7]. Among Saudi men, the prevalence of osteoporosis reached approximately 31 percent. Local studies have also indicated a notable burden of osteoporosis-related fractures, including vertebral fractures with reported rates between 20 and 24 percent [8-10]. Most hospital-based data suggest that femoral fractures are increasing, particularly among older adults, and that many patients remain undiagnosed or untreated for underlying osteoporosis [11].

Although fragility fractures can occur at multiple skeletal sites, hip and femoral fractures represent the most clinically severe and health-system-intensive outcomes of osteoporosis [2]. These fractures are associated with the highest rates of mortality, functional decline, loss of independence, and healthcare utilization, and they almost invariably require hospitalization and surgical intervention [2]. Consequently, hip and femoral fractures are widely used as sentinel events in osteoporosis research and health-service planning, enabling meaningful comparison with international benchmarks and quality-of-care indicators [5].

As life expectancy continues to increase in Saudi Arabia, the number of individuals at risk of fragility fractures will grow accordingly. Understanding the epidemiology, risk factors, clinical outcomes, and health-system gaps specific to hip and femoral fractures within the Saudi context is therefore essential for informing national strategies aimed at prevention, early detection, timely surgical care, and improved post-fracture management.

## Review

Materials and methods

Study Design

This systematic review aimed to evaluate the epidemiology, risk factors, clinical outcomes, management patterns, and health-system gaps related to fragility hip and femoral fractures in Saudi Arabia. The review was conducted in accordance with the Preferred Reporting Items for Systematic Reviews and Meta-Analyses (PRISMA) 2020 statement and guided by the Cochrane Handbook for Systematic Reviews of Interventions [12,13].

The review question was structured using the PICO framework [14]: adults aged 50 years and older in Saudi Arabia (Population), exposure to fragility hip or femoral fractures (Intervention/Exposure), comparisons across fracture characteristics or management approaches where applicable (Comparison), and outcomes including epidemiology, risk factors, mortality, complications, quality of life, osteoporosis diagnosis and treatment, and health-system factors (Outcomes).

Search Strategy

A comprehensive literature search was conducted in four major databases: PubMed, Scopus, Web of Science, and Google Scholar, covering the period from database inception to October 2025. The search strategy combined keywords and Medical Subject Headings (MeSH) related to fragility fractures, hip fractures, femoral fractures, osteoporosis, morbidity, mortality, and Saudi Arabia. Search terms included combinations of “hip fracture,” “femoral fracture,” “fragility fracture,” “osteoporotic fracture,” “osteoporosis,” “Saudi Arabia,” and related synonyms using Boolean operators. Only studies published in English and conducted on human subjects were considered. Reference lists of included articles were also manually screened to identify additional eligible studies. The search strategy was designed to capture all relevant epidemiological, clinical, and health-system data pertaining to fragility hip and femoral fractures in Saudi Arabia.

Selection Criteria

Studies conducted in Saudi Arabia that examined fragility fractures of the hip or femur among adults were eligible for inclusion. Studies were required to report at least one relevant outcome, including epidemiological measures, risk factors, mortality, complications, functional or quality-of-life outcomes, osteoporosis diagnosis or treatment patterns, clinician practices, or public awareness. A fragility fracture context was defined as fractures resulting from low-energy mechanisms (such as a fall from standing height) or studies explicitly examining osteoporotic or age-related fracture risk. Retrospective and prospective cohort studies, cross-sectional studies, registry analyses, and qualitative studies were included.

Studies were excluded if they focused primarily on high-energy trauma (e.g., road traffic accidents), isolated femoral shaft fractures in younger trauma populations, pediatric cases, experimental or laboratory research, case reports, conference abstracts, or studies without extractable data specific to hip or femoral fragility fractures. Trauma-dominant studies were included only if they provided separate analyses or findings relevant to older adults or fragility-related mechanisms.

Study Selection

**Figure 1:**
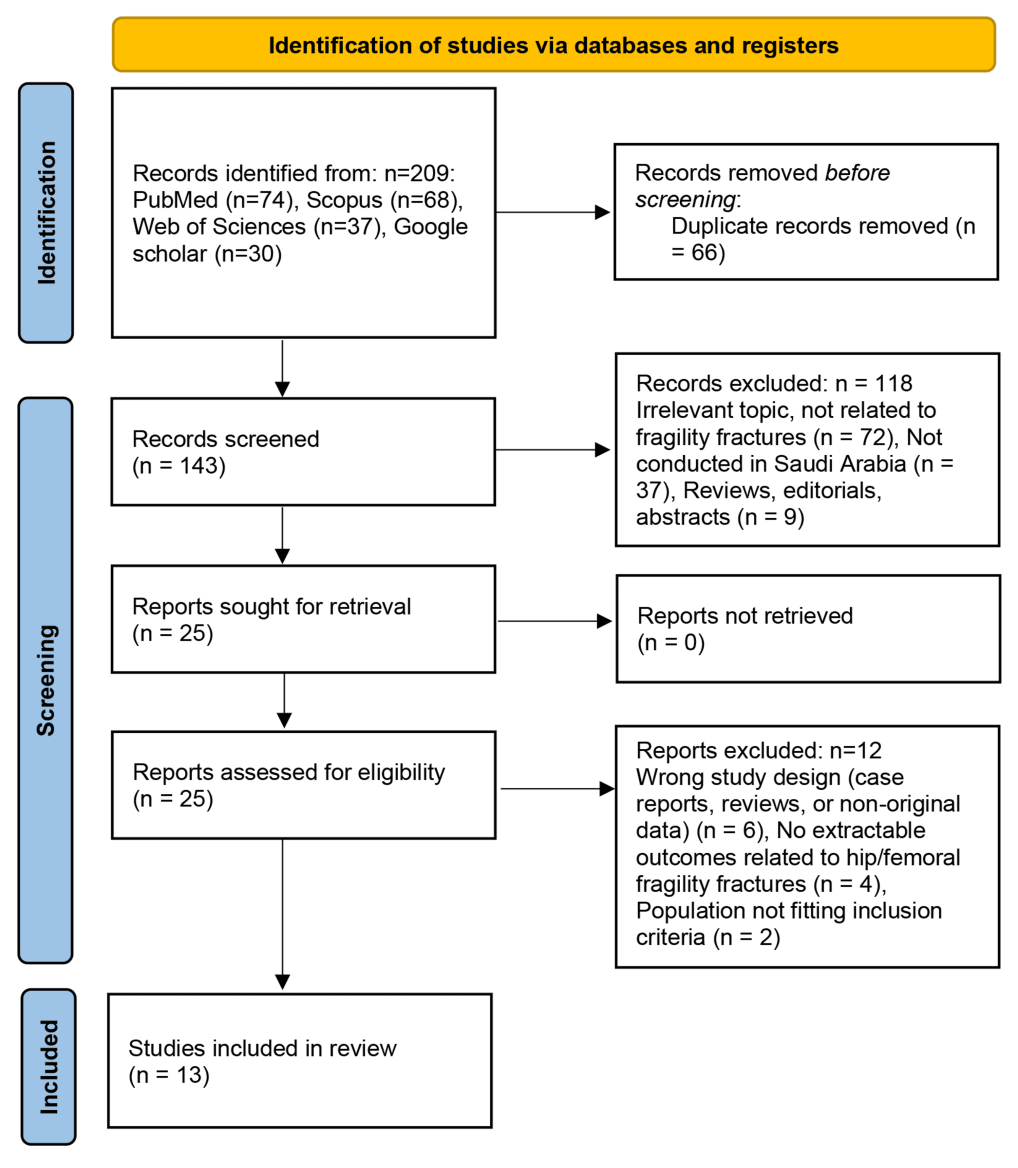
PRISMA flow diagram illustrating the selection process for studies included in the systematic review This flow diagram follows the PRISMA 2020 guidelines as described by Page et al. [13]. PRISMA: Preferred Reporting Items for Systematic Reviews and Meta-Analyses

All identified records were imported into Rayyan for duplicate removal [15]. Titles and abstracts were screened independently by six reviewers, and full texts of potentially eligible studies were assessed by four reviewers against the predefined inclusion and exclusion criteria. Discrepancies were resolved through discussion or consultation with a senior reviewer. The study selection process is summarized in the PRISMA flow diagram (Figure 1).

Data Extraction

Data were independently extracted by four reviewers using a standardized Excel form. Extracted variables included study characteristics (author, year, region, design, sample size, age, sex distribution), fracture type and prevalence, risk factors (e.g., age, osteoporosis, comorbidities, bone mineral density (BMD)), post-fracture outcomes (mortality, complications, quality of life), management patterns (osteoporosis diagnosis, treatment, dual-energy X-ray absorptiometry (DEXA) use), and health-system or clinician practice issues. When necessary, corresponding authors were contacted to clarify missing or unclear data. Extracted data were cross-verified to ensure accuracy and consistency.

Quality Assessment

The methodological quality of included observational studies was assessed using the Newcastle-Ottawa Scale (NOS) [16], which evaluates participant selection, comparability, and outcome ascertainment. Studies were categorized as high (7-9), moderate (4-6), or low quality (0-3). Assessments were performed independently by four reviewers, and any disagreements were resolved by consensus.

Data Synthesis

Given heterogeneity in study design, populations, and reported outcomes, no meta-analysis was performed. Findings were synthesized narratively, with key results summarized descriptively and presented in tables to facilitate comparison across studies and outcome domains.

Results

Study Selection

The database search identified 209 records across PubMed, Scopus, Web of Science, and Google Scholar. After the removal of 66 duplicates, 143 records underwent title and abstract screening. Of these, 118 were excluded mainly because they were unrelated to fragility fractures, not conducted in Saudi Arabia, or were review articles, editorials, or conference abstracts. Twenty-five full-text reports were retrieved and assessed for eligibility. Twelve studies were subsequently excluded due to unsuitable study design, absence of extractable outcomes specific to hip or femoral fragility fractures, or populations that did not meet the inclusion criteria. Thirteen studies met the eligibility criteria and were included in the final review. The selection process is summarized in the PRISMA flow diagram (Figure 1).

Characteristics of Included Studies

The 13 included studies were published between 2015 and 2025 and covered several regions within Saudi Arabia, including the Eastern Province, Riyadh, Jeddah, Madinah, Jazan, and Arar (Table 1). Most studies used retrospective designs, while four were cross-sectional and one was qualitative. Sample sizes ranged from 73 to 802 participants in hospital-based studies and up to 370 in community-based cohorts.

**Table 1:** Characteristics of the included studies BMD: Bone mineral density; DEXA: Dual-energy X-ray absorptiometry; FRAX: Fracture Risk Assessment Tool; HRQoL: Health-related quality of life; RTA: Road traffic accident; ASA: American Society of Anesthesiologists; NOF: Neck of femur; IT: Intertrochanteric. “Fragility fracture” refers to fractures resulting from low-energy mechanisms such as a fall from standing height. Studies with broader adult age ranges or unspecified age limits were included only when findings relevant to older adults or fragility-related hip or femoral fractures were reported or could be inferred from the study context. Trauma-dominant studies were retained solely for contextual epidemiological information or where fragility-relevant subgroups (e.g., neck of femur fractures in older adults) were identifiable and were not used to derive primary fragility risk estimates. Only studies conducted in Saudi Arabia and reporting extractable epidemiological, clinical, or outcome data were included. Study designs reflect the methodologies reported by the original authors.

Study (author, year)	Region/city	Study design	Setting	Study period	Population characteristics	Sample size	Fracture type/condition	Aim of the study	Notes
Sadat-Ali et al., 2015 [17]	Eastern Province (national extrapolation)	Retrospective	24 hospitals	2013	Adults ≥55	681 patients (780 fractures)	Proximal femur fractures	Estimate national incidence & economic burden	National modeling
Sadat-Ali et al., 2017 [11]	Al Khobar	Retrospective	King Fahd Hospital	2010–2014	Adults with proximal femur fractures	189	Fragility proximal femur fractures	Assess mortality & function	Includes ASA
Alanazi et al., 2018 [8]	Arar	Cross-sectional	Arar Central Hospital	2017–2018	Adults; 59.8% males	107	Hip fractures among others	Identify risk factors and complications	Includes postoperative complications
Sonbol et al., 2018 [18]	Madinah	Retrospective	King Fahad Hospital	2010–2016	Adults >16; mostly male	591	Femoral shaft fractures (mostly trauma)	Assess prevalence & injuries	Trauma-focused
Alfadhli et al., 2019 [19]	Madinah	Cross-sectional	Taibah Diagnostic Center	2016–2018	Women ≥45	370	Osteoporosis/osteopenia	Determine prevalence & FRAX risk	FRAX with & without BMD
Alsheikh et al., 2020 [20]	Riyadh	Retrospective	Tertiary hospital	2008–2018	Adults ≥60	802	Hip fractures	Identify mortality predictors	Includes complications & surgical delay
Sadat-Ali et al., 2020 [21]	Al Khobar	Retrospective	KFHU	2017–2018	Adults ≥50	187	Fragility fractures	Determine hospitalization rate & treatment gap	85.5% untreated
ALJohani et al., 2022 [22]	Riyadh	Retrospective cohort	King Saud Medical City	2010–2021	Adults 63–80	108	Proximal & distal femur fractures	Assess 1-year mortality & BMD effect	BMD paradox finding
Shehata et al., 2023 [23]	—	Cross-sectional	—	2023	Elderly	—	Femoral neck fracture knowledge	Assess public awareness	Public knowledge
El-Setouhy et al., 2024 [9]	Jazan	Cross-sectional	Community-based	2023	Postmenopausal women	158	Osteoporotic fracture history	Assess HRQoL & determinants	WHOQOL-BREF
Turabi et al., 2024 [10]	—	Qualitative	—	2024	Clinicians & stakeholders	—	Mobilization practices	Explore practice variability & barriers	Qualitative
Badghish et al., 2025 [24]	Jeddah	Retrospective	King Abdullah Medical Complex	2016–2021	Adults	73	Hip fractures	Identify 1-year mortality predictors	Surgical delay & ASA findings
Alharthy et al., 2025 [25]	Jeddah	Retrospective cohort	KAMC Jeddah	2004–2024	Adults ≥60	314	Fragility hip fractures	Examine diagnosis gaps & management	Very low DEXA use

Populations primarily included older adults, typically aged 50 years and above, with some studies focusing on postmenopausal women or elderly individuals. Hip fractures, proximal femur fractures, and general fragility fractures were the most frequently examined conditions. Two studies assessed osteoporosis prevalence and fracture risk, one evaluated public knowledge, and one explored clinician perspectives on mobilization practices. Study aims varied across epidemiological quantification, identification of risk factors, mortality assessment, evaluation of BMD, and exploration of management patterns.

Epidemiology and Burden of Disease

Across the included studies, fragility hip and femoral fractures emerged as a substantial and growing contributor to orthopedic admissions in Saudi Arabia, particularly among older adults (Table 2). National modeling data estimated more than 7,500 femoral fractures annually, with a considerable associated economic burden. Hospital-based cohorts consistently showed that femoral and hip fractures accounted for a large proportion of fragility-related admissions, underscoring their disproportionate impact on inpatient services.

**Table 2:** Outcomes and key findings from the included studies Findings represent extracted results as reported by each study and have not been statistically pooled. Mortality values correspond to the time frames specified in each study (e.g., 30-day, 1-year). Predictors refer to statistically significant associations identified through multivariate or univariate analyses. “High-risk fracture probability” follows FRAX thresholds (≥20% for major osteoporotic fracture, ≥3% for hip fracture). Postoperative complications were reported when applicable. Quality-of-life outcomes reflect WHOQOL-BREF domain scores. Studies with mixed fracture types were included only when femoral or hip fracture data were explicitly provided. DEXA: Dual-energy X-ray absorptiometry; NOF: Neck of femur; FRAX: Fracture Risk Assessment Tool; HRQoL: Health-related quality of life; BMD: Bone mineral density

Study	Outcome category	Key findings	Significant predictors/risk factors	Clinical implications
Sadat-Ali et al., 2015 [17]	National incidence & economic burden	~7,528 annual femur fractures; SR 564.75M annual cost; 82.1% untreated	Age	Large national healthcare burden; need prevention programs
Sadat-Ali et al., 2017 [11]	Mortality & function	1-year mortality 26.98%; only 48% regained pre-fracture mobility	ASA 4; poor pre-fracture mobility	Hip fractures carry worse outcomes than MI or stroke
Alanazi et al., 2018 [8]	Risk factors & complications	Hip fractures 22.4%; osteoporosis 64.3%; complications 33.3%	Age ≥60, osteoporosis, chronic illness, smoking	Screening and targeted prevention needed
Sonbol et al., 2018 [18]	Trauma epidemiology	Young men (78%); head injuries common; NOF fractures in older adults	Age; high-energy trauma	Useful for broader femoral injury burden
Alfadhli et al., 2019 [19]	Osteoporosis prevalence & FRAX	Osteoporosis 36.7%; hip FRAX ≥3% in 4.3%; higher FRAX risk without BMD	Age ≥70	FRAX without BMD could guide screening
Alsheikh et al., 2020 [20]	Mortality predictors	30-day mortality 4%, 1-year 11%; complications in 16%; delayed surgery ↑ mortality	NOF fractures, complications, surgery delay >48h	Importance of fast-track surgery and monitoring
Sadat-Ali et al., 2020 [21]	Hospitalization & treatment gap	Fragility fractures = 13.26% admissions; femur 72.7%; 85.5% untreated	—	Major treatment gap in osteoporosis care
ALJohani et al., 2022 [22]	Mortality & BMD effect	1-year mortality 21.3%; highest mortality in normal BMD group (38.3%)	Diabetes, hypertension (in normal-BMD group)	Challenges assumptions about BMD and mortality
Shehata et al., 2023 [23]	Public awareness	Low awareness of femoral neck fractures in elderly	Low education levels	Need national education campaigns
El-Setouhy et al., 2024 [9]	HRQoL & fracture prevalence	39% had fractures; femur = 11%; vertebral fractures ↓ QoL	Older age, inactivity, vertebral fracture	Emphasizes rehab and long-term care needs
Turabi et al., 2024 [10]	Clinical practice variability	Clinicians report inconsistent mobilization; systemic barriers	Staffing, protocols, training gaps	Need standardized mobilization pathways
Badghish et al., 2025 [24]	Mortality predictors	1-year mortality 47.9%; NOF mortality 74%	ASA ≥3; surgery delay >48h; fracture type	High-risk patients need rapid surgical intervention
Alharthy et al., 2025 [25]	Diagnosis & management gaps	DEXA use only 16.6%; vitamin D & calcium use increased post-fracture	Female sex (OR 2.46); obesity (OR 2.12); DEXA (OR 22.2)	Post-fracture osteoporosis care remains inadequate

Although community-based studies reported fractures at multiple skeletal sites, femoral fractures represented a smaller but clinically significant proportion, particularly among postmenopausal women. Trauma-dominant studies were used cautiously and primarily to contextualize broader femoral fracture patterns; fragility-specific interpretations were drawn only where age-related or low-energy mechanisms were evident.

Risk Factors for Fragility Fractures

Several studies identified age as a consistent determinant of fracture occurrence and adverse outcomes (Table 2). Alanazi et al. [8] found that individuals aged 60 years and older were at significantly higher risk of hip fractures, particularly in the presence of comorbidities such as osteoporosis and chronic illness. Smoking, physical inactivity, and low BMD were additional contributors in specific cohorts. In the community-based study by El-Setouhy et al. [9], older age and decreased physical activity were associated with reduced quality of life and increased fracture prevalence among postmenopausal women.

Interestingly, ALJohani et al. [22] observed that mortality was highest among patients with normal BMD, a counterintuitive finding that highlights the complexity of fracture risk beyond BMD alone. Obesity and female sex were also reported as predictors of diagnosis and treatment utilization in the study by Alharthy et al. [25].

Clinical Outcomes

Mortality following hip and femoral fractures varied substantially across studies, with reported one-year mortality ranging from approximately 11 percent to nearly 48 percent. Despite this variability, delayed surgery, high ASA classification, and poor pre-fracture functional status consistently emerged as predictors of mortality. Postoperative complications were common and contributed to prolonged recovery and functional decline. Functional outcomes were generally poor, with fewer than half of patients regaining pre-fracture mobility at one year in studies that reported this outcome. Quality-of-life data, though limited, demonstrated sustained impairment among fracture survivors, particularly those with vertebral or multiple fractures.

Management Patterns and Health-System Gaps

The review revealed substantial gaps in osteoporosis diagnosis and secondary prevention. Across studies, between 64 and 85 percent of patients with fragility fractures had underlying osteoporosis or osteopenia, yet treatment initiation remained strikingly low. In the study by Sadat-Ali et al. (2020) [21], more than 85 percent of patients received no osteoporosis therapy following a fragility fracture. Similarly, Alharthy et al. (2025) [25] reported that DEXA scanning was performed in only 16.6 percent of patients despite clear clinical indications.

Clinical practice variability was highlighted in a qualitative study where clinicians described inconsistent mobilization practices, partly driven by staffing constraints, inadequate protocols, and differing interpretations of postoperative care recommendations. The potential utility of FRAX for screening was demonstrated by Alfadhli et al. (2019) [19], who found that fracture risk remained significant even when BMD was not considered.

Quality of Included Studies

The methodological quality of the included studies ranged from moderate to high based on the NOS (Table 3). Seven studies were rated as high quality, generally demonstrating strong selection methods, appropriate comparability, and reliable outcome ascertainment. The remaining six studies were rated as moderate quality, often due to limitations in design structure, sample representativeness, or outcome assessment. No studies were rated as low quality. These ratings provide confidence in the overall strength of the evidence while highlighting areas for improvement, particularly in the design of prospective and interventional research.

**Table 3:** Quality assessment of included studies using the Newcastle–Ottawa Scale (NOS) The Newcastle–Ottawa Scale (NOS) evaluates observational studies across three domains: Selection (maximum 4 points), Comparability (maximum 2 points), and Outcome/Exposure (maximum 3 points), for a total maximum score of 9. Quality ratings were categorized as high (7–9), moderate (4–6), or low (0–3). Scores were assigned independently by reviewers, with discrepancies resolved by consensus. The use and structure of the NOS are based on the methodological evaluation described by Stang et al. [16].

Study	Selection (Max: 4)	Comparability (Max: 2)	Outcome/Exposure (Max: 3)	Total score (Max: 9)	Quality rating
Sadat-Ali et al., 2015 [17]	4	2	3	9	High
Sadat-Ali et al., 2017 [11]	3	2	3	8	High
Alanazi et al., 2018 [8]	3	1	2	6	Moderate
Sonbol et al., 2018 [18]	3	1	2	6	Moderate
Alfadhli et al., 2019 [19]	4	2	3	9	High
Alsheikh et al., 2020 [20]	4	2	3	9	High
Sadat-Ali et al., 2020 [21]	3	1	2	6	Moderate
ALJohani et al., 2022 [22]	3	2	3	8	High
Shehata et al., 2023 [23]	3	1	2	6	Moderate
El-Setouhy et al., 2024 [9]	4	2	3	9	High
Turabi et al., 2024 [10]	3	1	2	6	Moderate
Badghish et al., 2025 [24]	3	2	3	8	High
Alharthy et al., 2025 [25]	4	2	3	9	High

Discussion

This systematic review synthesizes available evidence on the epidemiology, risk factors, clinical outcomes, management patterns, and health-system gaps related to fragility hip and femoral fractures in Saudi Arabia. Although the evidence base is limited and heterogeneous, the included studies consistently demonstrate that these fractures impose a substantial clinical and health-system burden, particularly among older adults. The findings also highlight important gaps in diagnosis, timely surgical care, and secondary prevention that are directly supported by Saudi-based data.

In line with international data showing marked increases in hip fracture incidence among aging populations [5], Saudi studies document a significant proportion of orthopedic admissions attributable to fragility fractures. Estimates from local cohorts suggest that femoral fractures constitute nearly three-quarters of fragility-related hospitalizations, reflecting trends similar to high-income countries where hip fractures remain the most clinically consequential osteoporotic fracture [26]. While global projections estimate more than 1.6 million hip fractures annually worldwide [26], the national modeling study included in this review estimates over seven thousand annual femoral fractures in Saudi Arabia, accompanied by a substantial economic burden [17]. These national estimates reinforce earlier work suggesting that osteoporosis prevalence in Saudi Arabia is comparable to or higher than many Western countries, where roughly one in three women and one in five men over 50 are affected [27].

Risk factor profiles among Saudi adults align with international findings while also illustrating region-specific patterns. Increasing age emerged consistently as a major determinant of hip and femur fractures, which is consistent with global literature showing exponential rises in hip fracture incidence after the age of 70 [28]. Osteoporosis and chronic illnesses were frequently identified as risk factors, comparable to global evidence linking low BMD, frailty, and multimorbidity with fracture risk [8]. Notably, smoking, physical inactivity, and low awareness levels featured prominently in some Saudi cohorts, reflecting lifestyle and health-education gaps unique to the regional context. The finding that individuals with normal BMD may still experience higher mortality, as seen in one included study, highlights the complexity of post-fracture trajectories and supports international observations that frailty, sarcopenia, and comorbidities contribute substantially to outcomes beyond bone density alone [11,24,29].

Mortality following hip and femoral fractures varied widely across Saudi studies, with reported one-year mortality ranging from approximately 11 percent to nearly 48 percent. This variation likely reflects differences in patient age, fracture type, comorbidity burden, ASA classification, surgical timing, and study design. Despite this heterogeneity, delayed surgical intervention emerged as one of the most consistent predictors of mortality across multiple cohorts. Several studies identified delays beyond 48 hours as significantly associated with worse outcomes, reinforcing international evidence that timely surgery is a critical determinant of survival [20]. High ASA classification and postoperative complications were also associated with increased mortality, consistent with findings from multinational studies [30]. Together, these results emphasize that potentially modifiable system-level factors play a major role in patient outcomes.

One of the most robust findings of this review is the persistent gap in osteoporosis diagnosis and secondary prevention following fragility fractures in Saudi Arabia. Across multiple studies, more than 80 percent of patients did not receive osteoporosis treatment after fracture, and DEXA scanning was performed in fewer than 20 percent of cases. These findings mirror the treatment gap reported internationally [31,32], but the consistency of underdiagnosis and undertreatment across Saudi studies indicates a significant local health-system challenge. Limited access to DEXA scanning, variability in clinician awareness, patient-level barriers, and the absence of standardized post-fracture assessment pathways likely contribute to this gap. The use of FRAX without BMD, as reported in one included study, may offer a pragmatic approach to initial risk stratification in settings where imaging access is limited, consistent with global recommendations [33].

Health-system and practice-related barriers were further highlighted in qualitative evidence, which identified variability in mobilization practices, staffing constraints, and inconsistent protocol implementation across institutions [10]. Although limited in scope, these findings align with the observed variability in clinical outcomes and underscore the need for standardized care pathways. International models such as orthogeriatric care, early mobilization protocols, and fracture liaison services have demonstrated effectiveness in improving outcomes [3,34]. The findings of this review suggest that adapting such models to the Saudi healthcare context could address several of the identified gaps.

Taken together, fragility hip and femoral fractures in Saudi Arabia share many epidemiological and clinical characteristics with global patterns, but the available local evidence also points to distinct challenges related to delayed surgery, underutilization of osteoporosis care, and variability in clinical practice. As the Saudi population continues to age, the burden of these fractures is likely to increase unless targeted national strategies are implemented. Priorities should include improving access to bone health assessment, ensuring timely surgical management, standardizing postoperative care pathways, and strengthening secondary fracture prevention services.

Limitations

This review is limited by heterogeneity in study design, sample size, and outcome reporting across the included studies, which precluded meta-analysis and limited quantitative comparison. Most available evidence was derived from retrospective, single-center, hospital-based studies, which may introduce selection bias and restrict generalizability to the broader Saudi population. In addition, national fracture burden estimates and some key associations, such as mortality predictors, were informed by a small number of studies and should therefore be interpreted cautiously. Regional representation was uneven, and several important outcomes, including long-term functional recovery, quality-of-life trajectories, and cost analyses, were inconsistently reported. Despite these limitations, this review provides a structured synthesis of the available Saudi evidence and identifies priority areas for future multicenter and prospective research.

## Conclusions

This systematic review indicates that fragility hip and femoral fractures constitute an important clinical and health-system challenge in Saudi Arabia, particularly among older adults. The available evidence, derived largely from hospital-based studies, suggests that these fractures are associated with substantial mortality, functional decline, and postoperative complications, with poorer outcomes consistently observed when surgical care is delayed or comorbidity burden is high. Despite the high prevalence of osteoporosis among fracture patients, screening and secondary prevention measures remain markedly underutilized. Although the evidence base is limited and heterogeneous, the included studies consistently identify gaps in timely surgical management, osteoporosis diagnosis and treatment, and standardized post-fracture care. Addressing these gaps through locally adapted strategies, including improved access to bone health assessment, timely surgical pathways, and structured secondary fracture prevention programs, may help reduce the long-term burden of fragility hip and femoral fractures in Saudi Arabia.
